# Assessing the effectiveness of greater occipital nerve block in chronic migraine: a systematic review and meta-analysis

**DOI:** 10.1186/s12883-024-03834-6

**Published:** 2024-09-07

**Authors:** Muhamad Saqlain Mustafa, Shafin bin Amin, Aashish Kumar, Muhammad Ashir Shafique, Syeda Mahrukh Fatima Zaidi, Syed Ali Arsal, Burhanudin Sohail Rangwala, Muhammad Faheem Iqbal, Adarsh Raja, Abdul Haseeb, Inshal Jawed, Khabab Abbasher Hussien Mohamed Ahmed, Syed Muhammad Sinaan Ali, Giustino Varrassi

**Affiliations:** 1 Department of Medicine, Jinnah Sindh Medical University, Karachi, 75510 Pakistan; 2 Department of Medicine, Shaheed Mohtarma Benazir Bhutto Medical College, Karachi, 75400 Pakistan; 3 Department of Medicine, Dow University of Health Science, Karachi, 74200 Pakistan; 4 Fondazione Paolo Procacci, Roma, 00193 Italy; 5 Faculty of Medicine, University of Khartoum, Khartoum, 11111 Sudan; 6 Department of Medicine, Liaquat National Hospital and Medical College, Karachi, Pakistan; 7 Jinnah Sindh Medical University, Rafiqi H J Shaheed Road, Karachi, 75510 Pakistan; 8 Shaheed Mohtarma Benazir Bhutto Medical College, Lyari Hospital Rd, Rangiwara Karachi, Karachi, 75400 Pakistan; 9 Dow University of Health Sciences, Mission Rd, New Labour Colony Nanakwara, Karachi, 74200 Pakistan; 10 Liaquat National Hospital & Medical College, Stadium Road, Karachi, 74800 Pakistan

**Keywords:** Chronic migraine, Greater occipital nerve block (GONB), Local anesthetics, Headache, Chronic migraine

## Abstract

**Background & aims:**

Chronic migraine poses a global health burden, particularly affecting young women, and has substantial societal implications. This study aimed to assess the efficacy of Greater Occipital Nerve Block (GONB) in individuals with chronic migraine, focusing on the impact of local anesthetics compared with placebo.

**Methods:**

A meta-analysis and systematic review were conducted following the PRISMA principles and Cochrane Collaboration methods. Eligible studies included case-control, cohort, and randomized control trials in adults with chronic migraine, adhering to the International Classification of Headache Disorders, third edition (ICHD3). Primary efficacy outcomes included headache frequency, duration, and intensity along with safety assessments.

**Results:**

Literature searches across multiple databases yielded eight studies for qualitative analysis, with five included in the final quantitative analysis. A remarkable reduction in headache intensity and frequency during the first and second months of treatment with GONB using local anesthetics compared to placebo has been reported. The incidence of adverse events did not differ significantly between the intervention and placebo groups.

**Conclusion:**

The analysis emphasized the safety and efficacy of GONB, albeit with a cautious interpretation due to the limited number of studies and relatively small sample size. This study advocates for further research exploring various drugs, frequencies, and treatment plans to enhance the robustness and applicability of GONB for chronic migraine management.

## Introduction

Among headache disorders, migraine is particularly ranked second worldwide in terms of disability and is the leading cause of disability among young women, according to the Global Burden of Disease 2019 data [1]. Recent findings indicate that the global prevalence of migraine is approximately 15%, which translates to 4.9% of all ill health measured in years lived with disability (YLDs) [2]. Women are more likely to experience migraine than men, particularly those aged 15–49 years [3]. Migraine has a substantial societal and financial impact owing to both direct and indirect costs resulting from decreased productivity and missed work [4].

Migraine is a complex neurovascular disorder that affects sensory processing and is characterized by a range of symptoms, with headache being the most common symptom [5]. Chronic migraine (CM) is defined as the frequent occurrence of headache episodes, with at least 15 or more episodes (which, on at least 8 days/month, have the features of migraine headache) occurring per month for more than three months [6]. Several medications are available for the preventive treatment of migraine, including anticonvulsants, antidepressants, beta-blockers, calcium channel blockers, botulinum toxin A, and more recently, drugs that block the calcitonin gene-related peptide (CGRP) pathway (i.e., monoclonal antibodies and antagonists) [7]. Despite the potential of anti-CGRP monoclonal antibodies (mAbs) in managing chronic migraine, a remarkable proportion of patients do not respond to this treatment [8]. Approximately 25% of patients are unresponsive to anti-CGRP monoclonal antibodies [9].

An important component of the brainstem, the Trigeminocervical Complex (TCC) acts as a central processing unit for pain and sensory data from the head and neck. This is the point of convergence of the upper cervical spinal nerves and the trigeminal nerve, which supplies feeling to the face, head, and some regions of the neck [10, 11].

One of the TCC’s primary functions is the confluence of the occipital and trigeminal nerves there. The trigeminal nerve transmits sensory data from the face, scalp, and meninges through its three main branches (ophthalmic, maxillary, and mandibular). In the meanwhile, feelings from the back of the head are transmitted by the occipital nerves, which originate from the upper cervical spinal roots [10, 11]. Wide-ranging integration of sensory inputs from the head and neck is made possible by the network formed when these neurons converge at the TCC. The brainstem area known as the trigeminocervical complex is crucial to migraine pain processing since it is responsible for processing pain signals originating from the head and neck. [10, 11]. The face, head, and neck region’s sensory data—especially pain—are integrated by the TCC. Because of this integration, the TCC is an important piece of the migraine jigsaw when it comes to interpreting the location and degree of pain. The trigeminal, occipital, and TCC nerves are intricately intertwined with one another. A series of neurological events are set off during a migraine episode, beginning with the stimulation of the trigeminal nerve. This activation increases pain signals by causing the production of inflammatory chemicals around the TCC and blood arteries in the brain [10, 11]. Accompanying this, the occipital nerves may also be affected, particularly if the headache radiates to the rear of the head. Because of its connection, the TCC is further stimulated by pain signals from the occipital area, worsening the migraine sensation (it produces a feedback loop) [10, 11].

The main sensory nerve that serves the occipital region is the Greater Occipital Nerve (GON), which predominantly originates from the C2 dorsal root. The GON block is used in acute and preventive headache treatments as it targets the anatomical and functional connections between the trigeminal and cervical fibers within the trigemino-cervical complex (TCC). The rationale for using GON blocks is based on the integration of sensory neurons from C2 in the upper cervical spinal cord with neurons in the trigeminal nucleus caudalis (TNC). However, the precise mechanisms by which GON blocks may affect the TCC and potentially reduce its activity are still being researched [12]. However, there is currently no standard protocol for GONB. Local anesthetics function by preventing the activation of voltage-gated sodium channels, which reduce the transmission of sensory signals originating from areas innervated by the greater occipital nerve, such as the medial region of the posterior scalp [13, 14], thereby preventing the activation of convergent neurons in the trigeminal-cervical complex. Combination therapy with corticosteroids may reduce inflammation, thereby attenuating pain, however, this role of corticosteroids also seems to be under debate.

The current management of chronic migraines is inadequate, as it lacks clear guidelines despite the various treatment options available. The evidence supporting the efficacy of GONB in preventing chronic migraines is limited and not recent [15–17]. However, the emergence of new clinical trials offers a promising opportunity for this study to provide valuable insights to healthcare providers. This study aims to fill the knowledge gaps by conducting a comprehensive systematic review and meta-analysis, providing healthcare professionals with a more complete understanding of the collective results of this approach for the treatment of chronic migraines.

## Methods

A meta-analysis and a comprehensive systematic review were conducted to assess the efficacy of GONB in patients with CM, adhering to Preferred Reporting Items for Systematic Reviews and Meta-Analyses (PRISMA) guidelines [18]. The PICO framework, a cornerstone of evidence-based medicine, organizes clinical questions and study designs into Population, Intervention, Comparison, and Outcome. In our research on chronic migraine treatment, we examine the efficacy of greater occipital nerve block (Intervention) with local anesthetics alone versus a placebo (Comparison) among adults with chronic migraine (Population), focusing on changes in migraine intensity measured by VAS, frequency, and adverse effects (Outcome).

### Eligibility criteria

Inclusion criteria for studies considered in this meta-analysis encompassed randomized controlled trials (RCTs) evaluating the efficacy of greater occipital nerve block (GONB) with local anesthetics alone compared to a placebo in adult individuals diagnosed with chronic migraine. Studies were required to report outcomes including changes in migraine intensity measured by Visual Analog Scale (VAS), frequency of migraine episodes, and documentation of adverse effects. Exclusion criteria comprised studies that incorporated corticosteroids in conjunction with local anesthetics for GONB, non-randomized or non-controlled trials, studies with insufficient data for outcome assessment, and those involving populations other than adults with chronic migraine.

### Endpoints

The primary efficacy endpoints were the change in headache intensity as measured by any scale, the frequency of headache (days per month) in the intervention group compared to the placebo group at a specific point in time, and the intensity of headache in the intervention group compared to the placebo group. To assess safety, the analysis focused on the number of participants who experienced at least one adverse event (AE) and the total number of participants who experienced AEs.

### Literature search and study selection

**Fig. 1:**
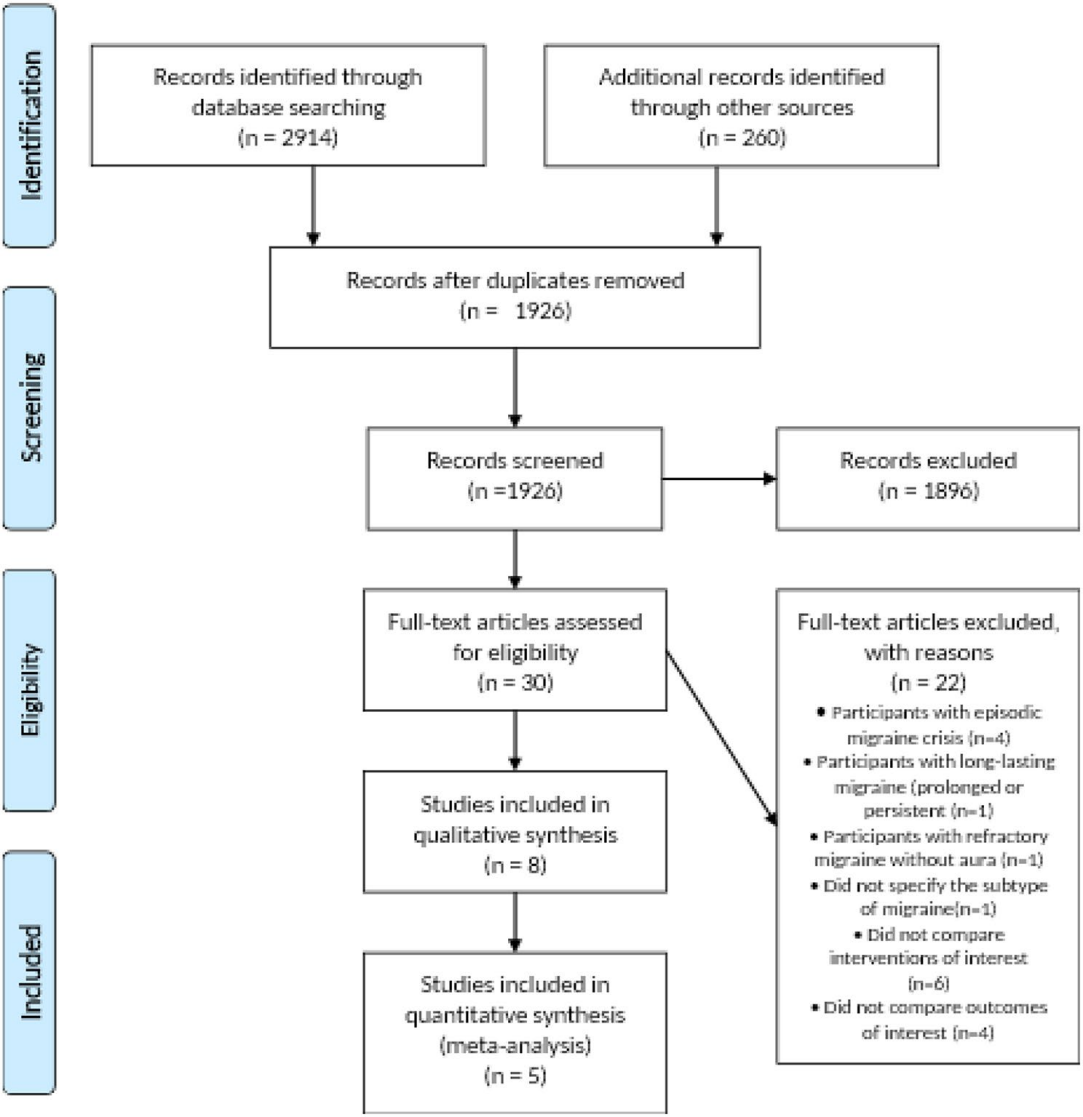
Prisma flow chart

A systematic search of PubMed, Medline, Scopus, Embase, Cochrane, Web of Science, and PsycINFO was performed as of June 2023 by two authors AR and AH. All languages and publication dates were considered and the search strategy involved both free and restricted terms pertaining to migraine and GONB, using key word ‘Chronic migraine’ or Migraine’ or ‘Greater Occipital Nerve Block’. Duplicates were eliminated and the titles and abstracts of the remaining articles were assessed to identify relevant studies. Subsequently, a full-text assessment was performed by two independent investigators (AK and BSR) and any discrepancies were resolved by a third investigator (MSM). The PRISMA flowchart (Fig. 1) illustrates the selection process.

### Data extraction

We utilized a standard Microsoft Excel 2021 spreadsheet to gather data from each study included in a predetermined format. Two unbiased investigators (MAS and SMFZ) collected the following information from each study: author, year of publication, population, intervention and comparison drugs, techniques, primary and secondary outcomes, funding and potential conflicts of interest. If a disagreement arose, a third investigator made the final decision (GV).

### Statistical analysis

Statistical analysis was conducted using Review Manager 5.3.22 and Comprehensive Meta-analysis. In order to account for anticipated between-study heterogeneity, we employed random-effects models in our meta-analysis of continuous outcomes. We reported the effect sizes as weighted mean differences (MD) with 95% confidence intervals (CI) for trials with similar results. The I^2^ statistics were used to assess the statistical heterogeneity of the pooled estimates. While recognizing that statistical heterogeneity may not be significant when I^2^ is < 40%, we performed this test. Regrettably, due to the limited number of included papers, we were unable to carry out a subgroup analysis or funnel plot assessment of publication bias.

## Results

### Studies selection

The initial literature search yielded 3174 studies. After a detailed review of the selected studies and removal of duplicate entries, 1964 articles remained. These articles were then evaluated based on their titles and abstracts to determine whether they met the inclusion criteria for our study and those that did not were excluded. A comprehensive screening of the full text was performed in the remaining 30 studies. Studies which did not meet the inclusion criteria were excluded. The final quantitative analysis included five studies and 3 studies were included in the qualitative assessment as these studies used other drugs like corticosteroids thus with different interventions. A visual representation of the PRISMA flowchart effectively illustrated the study selection process (Fig. 1).

### Quality assessment

**Fig. 2:**
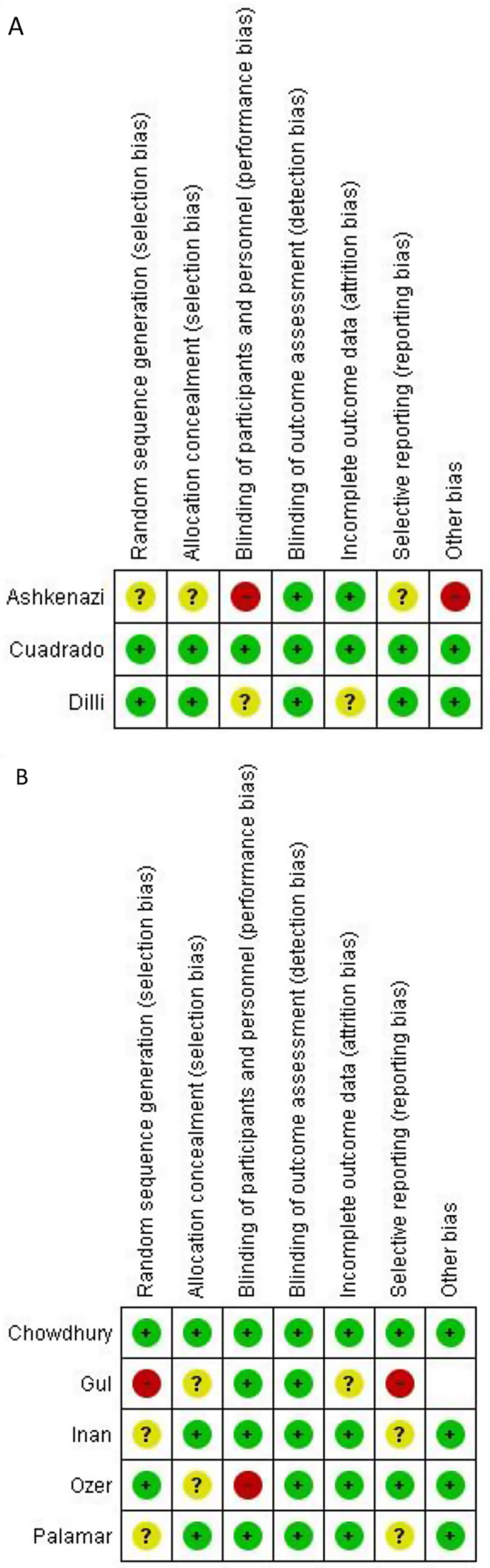
Risk of bias Assessment (**A**) Qualitative (**B**) Quantitative

In assessing the quality of RCTs, we extensively utilized the Cochrane Risk of Bias tool which categorizes studies into three risk levels: high, uncertain, and low, across seven specific domains encompassing aspects of selection, comparability, and outcome. Following rigorous evaluation, all studies included in our analysis were consistently classified as having low risk across these domains. A detailed presentation of the Risk of Bias assessment is shown in Fig. 2.

### Study and patient characteristics

All the included studies assessed outcomes in patients aged 18–75 years. The intervention group in three studies [19–21] used bupivacaine 0.5% 1.5 ml with or without 1 ml of saline (0.9%); one study [22] used lidocaine 2% 1 ml with 1 ml of saline solution (0.9%); and lastly, one study [23] used lidocaine 2% 2 ml as the interventional group. In the control groups, a saline solution of 0.9% (1.5, 2, or 2.5 ml) was used as a placebo. A total of 268 patients were included in all studies, ranging in age from 18 to 75 years. The studies differed in their follow-up procedures. Two studies were followed up at 4 weeks, one study was followed up for up to 2 months, and two studies were checked every month for up to 3 months A summary of patients’ baseline characteristics is provided in Table 1.

**Table 1:** Baseline characteristics of the patients

Study	Country	Participants	Diagnostic criteria	Intervention	Comparision
Cuadrado et al.	Spain	Women with chronic migraine (*n* = 36)	ICHD-3	Bupivacaine 0.5% 2 ml (*n* = 18)	Saline solution 0.9% 2 ml (*n* = 18)
Gul et al.	Turkey	Participants of both genders with chronic migraine (*n* = 44)	ICHD-2	Bupivacaine 0.5% 1.5 ml + 1 ml of saline 0.9% (*n* = 22)	Saline solution 0.9% 2.5 ml (*n* = 22)
Inan et al.	Turkey	Participants of both genders with chronic migraine (*n* = 72)	ICHD-2	Bupivacaine 0.5% 1.5 ml + 1 ml saline 0.9%. (*n* = 33))	Saline solution 0.9% 2.5 ml (*n* = 22
Dilli et al.	United States	Patients of both genders with episodic and chronic migraine (*n* = 70)	ICHD-2	Bupivacaine 0.5% 2.5 ml + Methylprednisolone 20 mg 0.5 ml (*n* = 33)	Lidocaine 1% 0.25 ml + Saline solution 0.9% 2.5 ml (*n* = 30)
Özer et al.	Turkey	Participants of both genders with chronic migraine (*n* = 71)	ICHD-3	Lidocaine 2% 1 ml + Saline solution 0.9% 1 ml (*n* = 17)	Saline solution 0.9% 2 ml (*n* = 11)
Ashkenazi et al.	United States	Participants of both genders with chronic migraine (*n* = 37)	ICHD-2	Lidocaine 2% 4.5 ml + Bupivacaine 0.5% 4.5 ml + Triamcinolone 40 mg/mL (*n* = 18)	Lidocaine 2% 4.5 ml + Bupivacaine 0.5% 4.5 ml + Saline solution 0.9% 1 ml (*n* = 19)
Palamar et al.	Turkey	Participants of both genders with chronic migraine (*n* = 37)	ICHD-2	Bupivacaine 0.5% 1.5 ml (*n* = 11)	Saline solution 0.9% 1.5 ml (*n* = 12)
Chowdhury et al.	India	Participants of both genders with chronic migraine (*n* = 44)	ICH-3	Lidocaine 2% 2 ml (*n* = 22)	Saline solution 0.9% 2 ml (*n* = 22

### Outcome

#### Effect of GONB on headache intensity

**Fig. 3:**
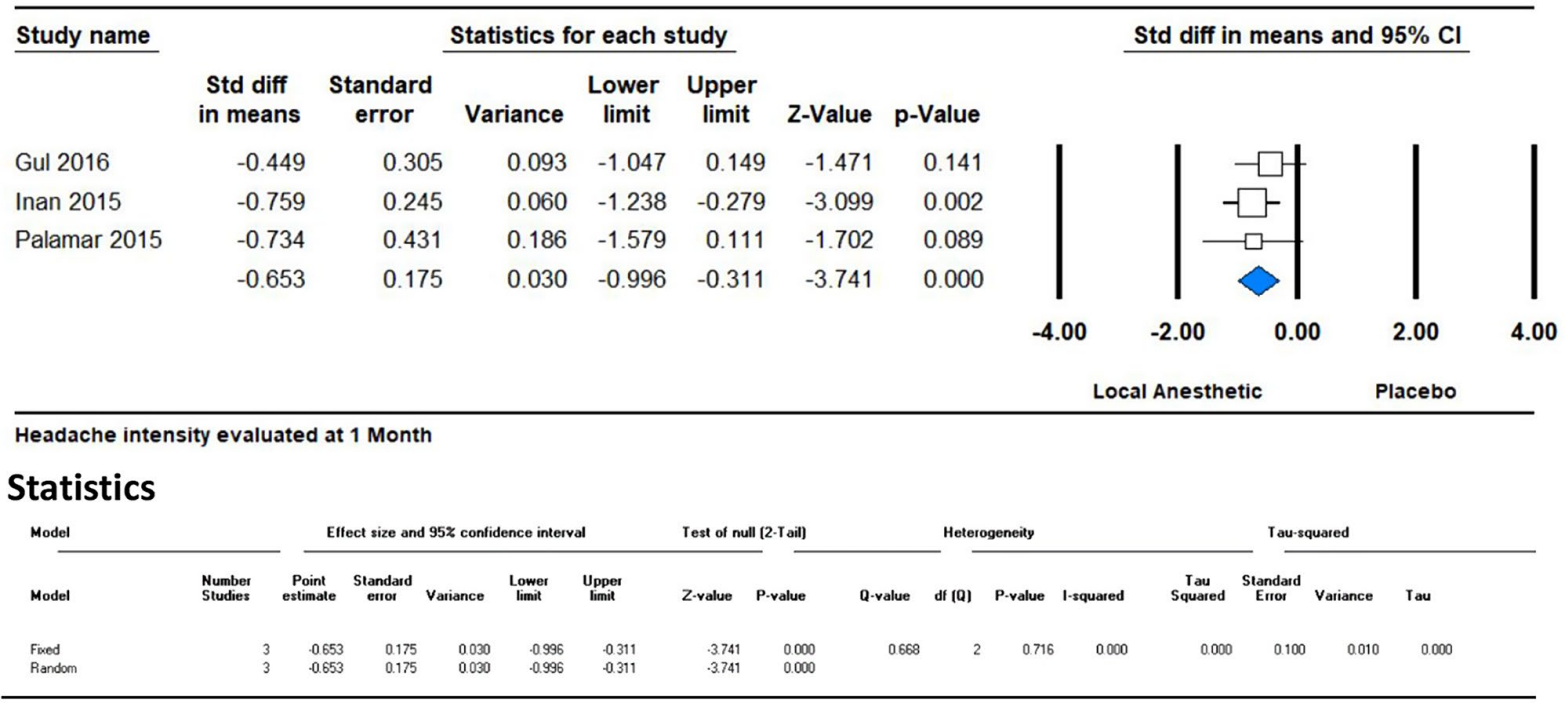
Forest plot illustrating the effect of GONB on headache intensity, evaluated using VAS within the initial month

In the initial month following GONB treatment, the meta-analysis of three studies showed a significant reduction in headache intensity as measured by the Visual Analog Scale (VAS). The standardized mean difference (SMD) was − 0.653, with a 95% confidence interval (CI) of -0.996 to -0.311 and a p-value of 0.0001. This indicates that the local anesthetic group experienced a greater reduction in headache intensity compared to the placebo group. Importantly, the I² value of 0% suggests that there was no observed heterogeneity among the studies, indicating consistent results across the studies analyzed. (Fig. 3)

**Fig. 4:**
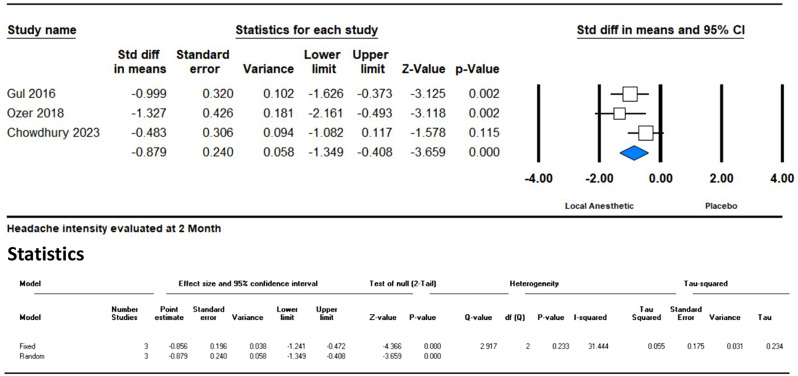
Forest plot illustrating the impact of GONB on headache intensity, evaluated using VAS during the second month

**Fig. 5:**
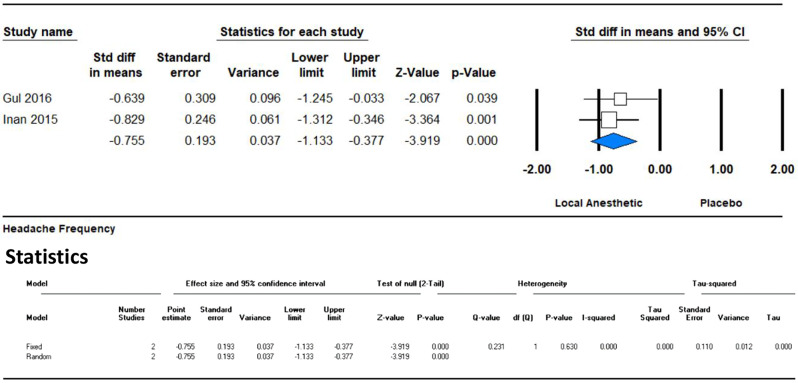
Forest plot illustrating the effect of GONB on headache frequency within the initial month

In the second month, an analysis of five studies continued to show a significant reduction in headache intensity with an SMD of -0.628 (95% CI -1.148 to -0.107; *p* = 0.018). However, the I² value increased to 74%, indicating substantial heterogeneity among the studies. This heterogeneity was primarily due to one study (Inan et al.), which had an outlier SMD of 0.136. (Fig. 4) A leave-one-out analysis was conducted to address this issue and is shown in Fig. 5.

#### Headache frequency

**Fig. 6:**
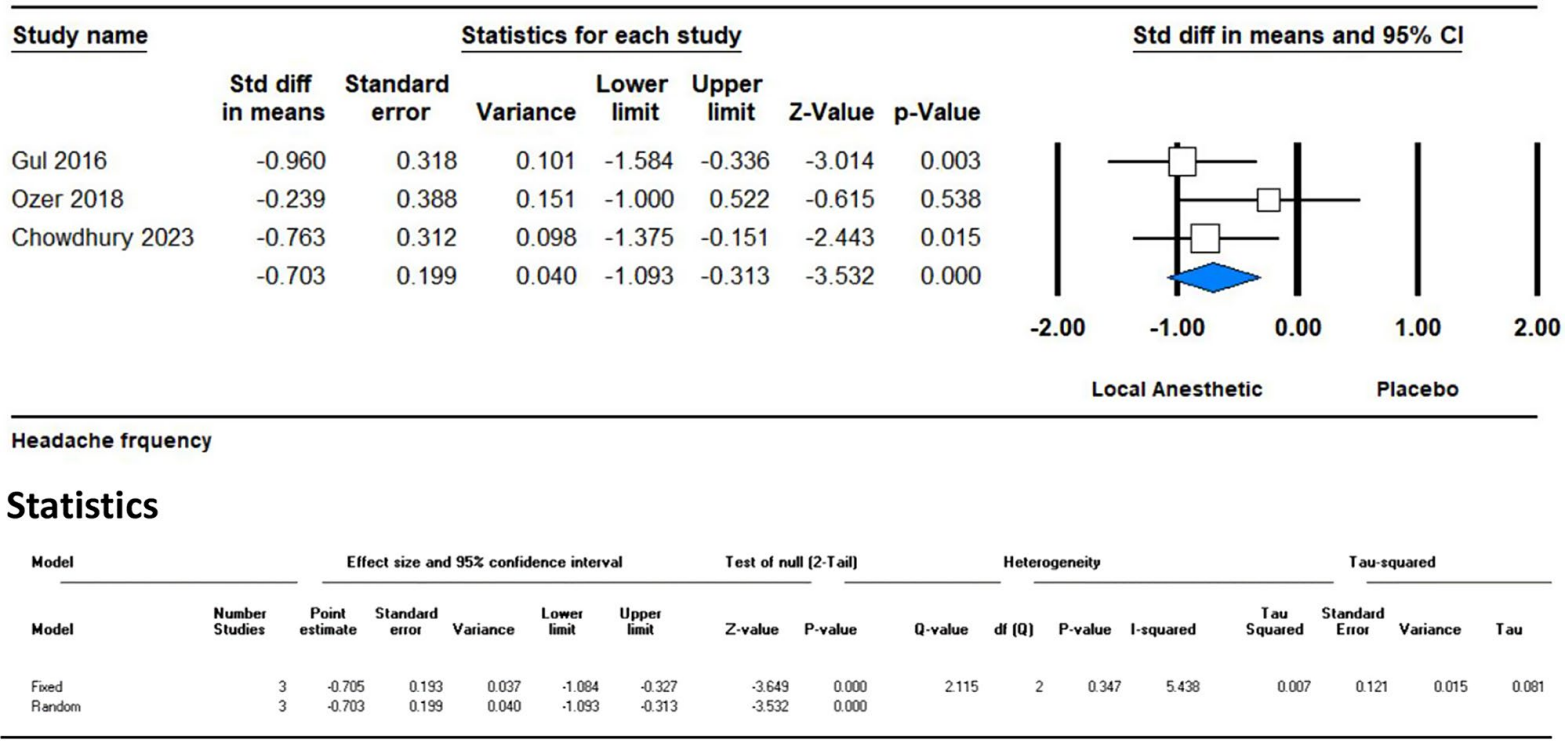
Forest plot illustrating the impact of GONB on headache frequency during the second month

Within the initial month, the analysis of two studies showed a significant reduction in headache frequency, with an SMD of -0.755 (95% CI -1.133 to -0.377; *p* = 0.0001). The results indicate a notable decrease in headache frequency in the local anesthetic group compared to the placebo group. The I² value of 0% indicates no heterogeneity between the studies, suggesting that the results were consistent. (Fig. 6)

**Fig. 7:**
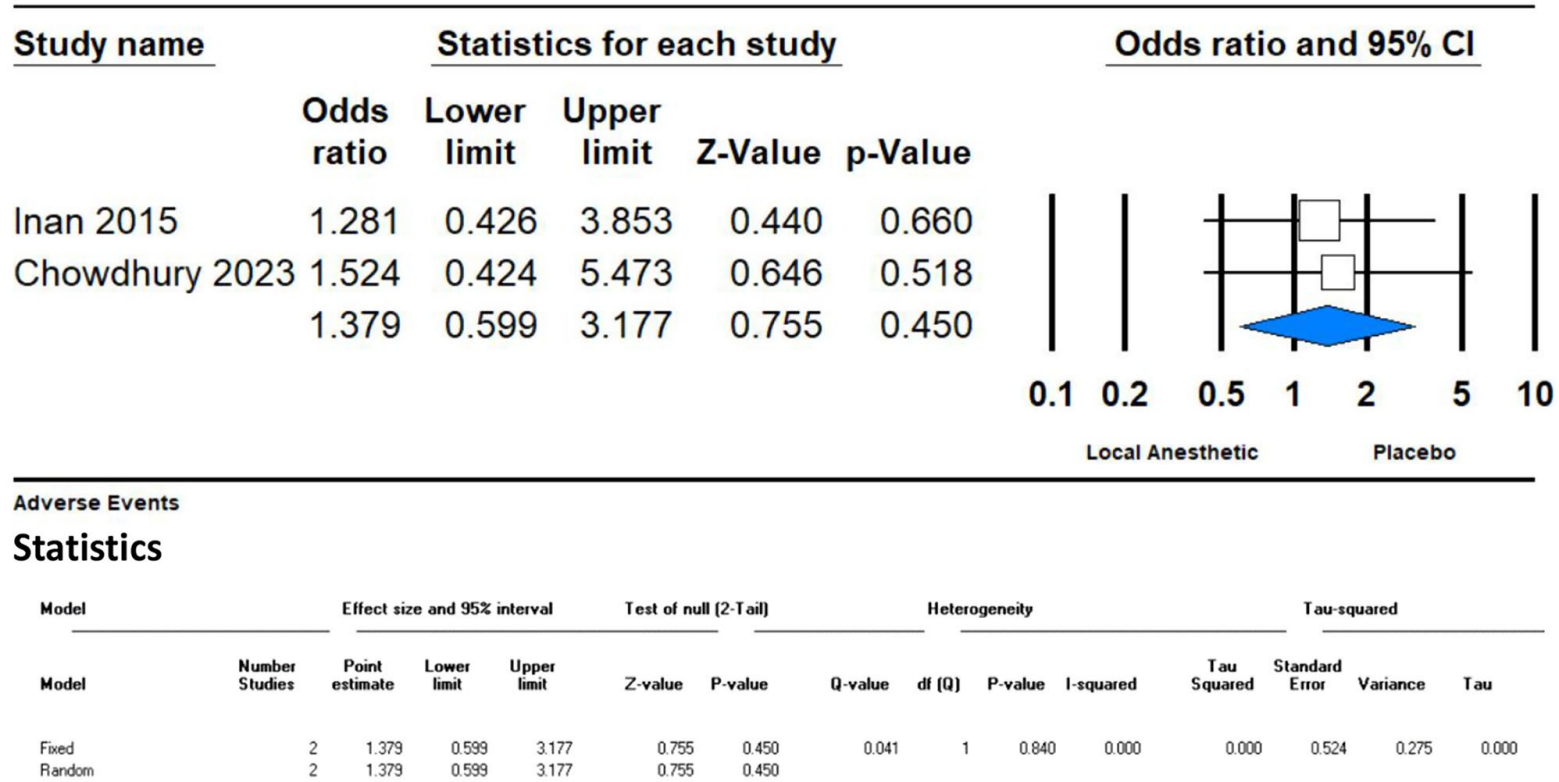
Forest plot displaying adverse events associated with the use of GONB

At the two-month mark, the analysis of four studies also showed a significant reduction in headache frequency with an SMD of -0.577 (95% CI -0.887 to -0.266; *p* = 0.0001). The low I² value of 8.9% indicates minimal heterogeneity among the studies, reinforcing the consistency of the observed effect (Fig. 7).

#### Adverse events

The meta-analysis of two studies on adverse events revealed no significant difference between the GONB treatment and placebo groups. The odds ratio (OR) was 1.379 with a 95% CI of 0.599 to 3.177 and a p-value of 0.450. The confidence interval crosses one, indicating that there is no clear increased risk of adverse events associated with GONB treatment. Additionally, the I² value of 0% suggests no heterogeneity between the studies, indicating consistent findings regarding the safety profile of GONB (Fig. 7).

## Discussion

We conducted an updated meta-analysis of GONB in patients with CM, incorporating findings from five RCTs. All RCTs used local anesthetics for GONB, while 0.9% saline served as the placebo. Our study focused on evaluating the impact of GONB on headache frequency, intensity, and associated adverse effects. The results demonstrated the beneficial effects of local anesthetics in reducing both the frequency and intensity of headaches during the first and second months of treatment. However, the outcomes related to adverse effects did not reach statistical significance. This meta-analysis included studies employing two distinct local anesthetics: 0.5% bupivacaine and 2% lidocaine. This suggests that the use of any local anesthetic could yield positive outcomes when compared with the effects of a placebo. Despite the positive results observed, we approached the evidence with caution because of the assessment of low certainty. Therefore, additional studies are warranted to further substantiate our findings and to enhance the reliability of the conclusions drawn from our meta-analysis.

Our meta-analysis demonstrated that GONB treatment significantly reduces both headache intensity and frequency in the initial and subsequent months post-treatment compared to placebo. During the first month, the studies consistently showed a marked reduction in headache intensity with no observed heterogeneity, indicating uniform results across the studies analyzed. In the second month, while the reduction in headache intensity remained remarkable, some heterogeneity was noted due to an outlier study. Similarly, the analysis revealed a notable decrease in headache frequency within the first month, again with consistent findings and no heterogeneity between the studies. By the second month, the reduction in headache frequency continued to be noteworthy, with minimal heterogeneity observed, reinforcing the consistency of the treatment effect. Furthermore, the analysis of adverse events indicated no significant difference between the GONB treatment and placebo groups, suggesting that GONB does not increase the risk of adverse events. The studies consistently supported the safety profile of GONB, with no observed heterogeneity. In terms of both safety and efficacy, our findings suggest that the use of local anesthetics in GONB is generally safe, as we did not identify any notable adverse effects in our intervention group. However, the certainty of our evidence is moderate, primarily because our results did not reach statistical significance, potentially influenced by the limited number of studies and relatively short follow-up phase. In our updated meta-analysis, building upon the original study by Velezquez et al. [24], we included an additional randomized RCT, contributing to a more comprehensive quantitative analysis. Although most of our study findings align with Velezquez’s findings [24], demonstrating the safety and effectiveness of GONB in treating chronic migraine, it is important to acknowledge some variations. Velezquez highlighted occasional negative effects associated with local anesthetics but found no remarkable side effects. In contrast, our study did not yield statistically significant outcomes in defining these results. A noteworthy distinction lies in the consideration of adjuvants: while our study did not account for steroids or other adjuvants, Velezquez considered steroids for every study outcome. This discrepancy underscores the need for further exploration and standardization of variables in future research to establish a more definitive understanding of the safety and efficacy of GONB in the management of chronic migraine.

Our findings strongly suggest that GONB is a safe and effective method for treating migraine. This assertion is consistent with existing research that characterizes GONB as a highly effective and safe therapy with minimal adverse effects, recommending its consideration when alternative treatments are unsuccessful [21]. This viewpoint is further supported by another study that affirms our findings, emphasizing a preference for GONB in cases of resistant migraine [22]. Moreover, evidence suggests the potential applicability of GONB in the treatment of various types of headaches [23, 25]. A retrospective cohort study also indicated that GONB may be beneficial in addressing acute migraine episodes, albeit with a cautionary note regarding the potential negative effects occurring during the procedure rather than during the follow-up period [26]. Additional observational studies [25, 27] reinforce our findings. However, a study comparing the effectiveness of GONB with placebo in preventing migraine revealed that while there was no marked change in headache frequency, GONB still played a remarkable role in lowering intensity [28]. Notably, these studies underscored the benefits of GONB, often involving the adjunct use of steroids. In a randomized controlled trial that focused on patients treated with bilateral GONB, the results indicated that the administration of a local anesthetic was associated with lower frequency, reduced intensity, and increased pressure thresholds. However, it is important to note that this study predominantly involved female participants [29]. However, it is essential to acknowledge that trials exclusively assessing the independent use of local anesthetics in GONB are currently lacking, as steroids are commonly employed as adjuvants in the majority of studies. This finding suggests the need for further investigation to delineate the unique contributions of local anesthetics to GONB outcomes.

Prior research has emphasized the necessity of comparing various treatment plans for GONB, incorporating diverse anesthetics and adjuncts to comprehensively evaluate its effectiveness, the need for additional intervention, and safety considerations, it is crucial to note that we did not incorporate any adjuncts, preventing us from commenting on their potential impact on the treatment outcomes. The absence of adjunct utilization in our study underscores the need for further exploration of how these additions may influence the overall efficacy and safety of GONB. Most trials in our analysis used weekly injections, resulting in a lack of comprehensive data for comparing various frequencies. Nevertheless, some studies have suggested the potential advantages associated with monthly use [26]. The American Headache Society also suggests and has shown interest in the efficacy of nerve blocks for headache treatment. Their endorsement highlights the growing recognition of nerve blocks as a valuable therapeutic option for managing headaches [30, 31].

Included studies present diverse methodologies in terms of dosage, injection sites, duration and timing of the intervention, and primary endpoints for the evaluation of GONB efficacy in migraine treatment. The administration and makeup of the GONB differed substantially across the studies. For example, Gul et al. [20] used 0.5% bupivacaine diluted in 1 ml, while Inan et al. [19] used a slightly larger volume of the same concentration. Ozer et al. [22] combined 2% lidocaine with saline, and Ashkenazi et al. [32] mixed lidocaine and bupivacaine. These variations could lead to differences in efficacy and side effects. The addition of corticosteroids, as observed in Dilli et al. [33], introduces another variable that may enhance the anti-inflammatory effects but could also influence the outcome independently of the nerve block’s anesthetic action. Although the studies targeted the GON, the exact injection sites varied slightly. Most studies, such as those by Gul et al. [20], Inan et al. [19], and Cuadrado et al. [34], selected a site approximately 2 cm lateral and 2 cm inferior to the external occipital protuberance. Palamar et al. [21] used ultrasound guidance, which might improve accuracy and potential efficacy. Ashkenazi et al. [32] included additional trigger point injections (TPIs), which could complicate the specific effects of the GONB.

The administration of GONB varied in frequency and duration among different studies. While some research, such as that conducted by Gul et al. [20] and Inan et al. [19], administered the blocks weekly for four weeks, others like Chowdury et al. [23] extended the injections over a period of 12 weeks. On the other hand, Cuadrado et al. [34] and Dilli et al. [33] examined single-time administrations. These discrepancies in timing may affect both short-term and long-term outcomes, with more frequent administrations potentially leading to more sustained relief, but also increasing the risks of cumulative side effects. The primary endpoints of the studies varied but generally included measures of headache frequency and intensity. For instance, Gul et al. [20] and Palamar et al. [21] focused on the number of headache days per month, while Inan et al. assessed both frequency and intensity. Ozer et al. [22] and Cuadrado et al. [34] emphasized the reduction in headache frequency, while Dilli et al. [33] sought a 50% reduction in migraine frequency as a measure of success. The variation in endpoints underscores the multifaceted nature of migraine impact and the significance of selecting appropriate, consistent measures for evaluating the efficacy of treatments.

Despite the differences in methodology, the studies collectively indicate that GONB can effectively decrease the frequency and severity of migraines. The consistent reporting of substantial improvements across a range of dosages, injection techniques, and primary outcomes reinforces the potential usefulness of GONB in clinical practice. However, the variation in methodologies highlights the need for standardized protocols to improve the comparability and generalizability of the findings. While the reviewed studies indicate promising outcomes for GONB in migraine treatment, the variability in dosage, injection sites, administration timing, and primary endpoints necessitates caution.

Examining these frequencies is particularly vital because of the invasive nature of the procedure, which offers valuable insights into its safety profile. An essential aspect of chronic migraine management is patient adherence, which markedly contributes to treatment success. It is imperative to assess the level of adherence to GONB. Unfortunately, we could not find relevant research on participants discontinuing their medication owing to side effects, hindering our ability to determine the tolerability of the treatment. Another unresolved concern revolves around the choice between unilateral and bilateral GONB and their relative efficacy. A retrospective cohort study comparing patients who underwent bilateral versus unilateral GONB demonstrated equal effectiveness [35]. However, a definitive conclusion remains elusive as additional evidence from diverse studies is lacking. Addressing these gaps in research would contribute substantially to refining our understanding of GONB’s optimal parameters for improved outcomes in chronic migraine management. Longitudinal studies and studies on the frequency of nerve block use are needed to assess long-term efficacy.

## Limitations

Although this meta-analysis offers valuable insights, it is crucial to acknowledge its limitations. First, the small sample size resulting from the limited availability of new studies may compromise the reliability and accuracy of our findings. Although incorporating more studies could alleviate this concern, the scarcity of available data remains an issue. Second, the absence of sufficient data from recent trials prevented consideration of baseline characteristics, hindering our ability to perform meta-regression. This limitation underscores the importance of comprehensive data collection in future studies to increase the depth of our analyses. Third, oversight of not accounting for pretreatment medications taken by patients during the procedure might introduce a confounding factor. Although the existing data may be insufficient to draw definitive conclusions, recognizing and addressing this aspect in future research is essential for a more nuanced understanding. Moreover, this meta-analysis did not explicitly address patient comorbidities. These factors could potentially influence the safety of the procedure in patients with various comorbidities. Future studies should delve into these aspects to provide a more comprehensive assessment of the safety profile of the procedure in diverse patient populations. In conclusion, although this meta-analysis provides valuable insights, researchers must remain cognizant of these limitations. Addressing these concerns in future studies will enhance the robustness and applicability of these findings in clinical settings.

## Conclusion

Based on our investigation, we ascertained that the administration of Greater Occipital Nerve Blocks (GONB) with local anesthetic leads to a notable reduction in both the intensity and frequency of headaches when compared to placebo. Additionally, our research underscores the effectiveness of GONBs and affirms their satisfactory safety profile. However, it is important to acknowledge that our confidence in these findings is somewhat tempered by the limited number of studies and relatively modest sample size that underpins our conclusions. Therefore, we advocate that future studies should broaden their scope by incorporating larger and more diverse sample sizes. These studies should also explore a range of drugs, frequencies, and treatment plans to augment the robustness and applicability of the results, thereby providing a more comprehensive understanding of the potential benefits of GONBs for headache management.

## Data Availability

The data are available within the article and supplementary files. The authors confirm that data supporting the findings of this study are available in the article and supplementary files.

## References

[1] Steiner TJ, Stovner LJ, Jensen R, Uluduz D, Katsarava Z. Migraine remains second among the world’s causes of disability, and first among young women: findings from GBD2019. J Headache Pain. 2020;21(1):137. 33267788 10.1186/s10194-020-01208-0 PMC7708887

[2] Steiner TJ, Stovner LJ. Global epidemiology of migraine and its implications for public health and health policy. Nat Rev Neurol. 2023;19(2):109–17. 36693999 10.1038/s41582-022-00763-1

[3] Stovner LJ, Nichols E, Steiner TJ, Abd-Allah F, Abdelalim A, Al-Raddadi RM, et al. Global, regional, and national burden of migraine and tension-type headache, 1990–2016: a systematic analysis for the global burden of Disease Study 2016. Lancet Neurol. 2018;17(11):954–76. 30353868 10.1016/S1474-4422(18)30322-3 PMC6191530

[4] Ferrari MD. The economic burden of migraine to society. PharmacoEconomics. 1998;13(6):667–76. 10179702 10.2165/00019053-199813060-00003

[5] Goadsby PJ, Holland PR. An update: pathophysiology of Migraine. Neurol Clin. 2019;37(4):651–71. 31563225 10.1016/j.ncl.2019.07.008

[6] The International Classification of Headache Disorders, 3rd edition (beta version). Cephalalgia. 2013;33(9):629–808. 10.1177/0333102413485658 23771276

[7] Urits I, Yilmaz M, Bahrun E, Merley C, Scoon L, Lassiter G, et al. Utilization of B12 for the treatment of chronic migraine. Best Pract Res Clin Anaesthesiol. 2020;34(3):479–91. 33004160 10.1016/j.bpa.2020.07.009

[8] Hong JB, Lange KS, Overeem LH, Triller P, Raffaelli B, Reuter U. A scoping review and Meta-analysis of Anti-CGRP monoclonal antibodies: Predicting Response. Pharmaceuticals (Basel). 2023;16(7). 10.3390/ph16070934 PMC10385131 37513846

[9] Han L, Liu Y, Xiong H, Hong P. CGRP monoclonal antibody for preventive treatment of chronic migraine: an update of meta-analysis. Brain Behav. 2019;9(2):e01215. 30656853 10.1002/brb3.1215 PMC6379644

[10] Al-Khazali HM, Krøll LS, Ashina H, Melo-Carrillo A, Burstein R, Amin FM et al. Neck pain and headache: Pathophysiology, treatments and future directions. Musculoskeletal Science and Practice [Internet]. 2023;66:102804. 10.1016/j.msksp.2023.102804 10.1016/j.msksp.2023.102804 37394323

[11] Bartsch T, Goadsby PJ. The trigeminocervical complex and migraine: current concepts and synthesis. Curr Sci Inc. 2003;7:371–6. 10.1007/s11916-003-0036-y. 10.1007/s11916-003-0036-y 12946290

[12] Chowdhury D, Datta D, Mundra A. Role of Greater Occipital nerve Block in Headache disorders: a narrative review. Neurol India. 2021;69(Supplement):S228–56. 34003170 10.4103/0028-3886.315993

[13] Anthony M. Headache and the greater occipital nerve. Clin Neurol Neurosurg. 1992;94(4):297–301. 1335856 10.1016/0303-8467(92)90177-5

[14] Selekler MH. [Greater occipital nerve blockade: trigeminicervical system and clinical applications in primary headaches]. Agri. 2008;20(3):6–13. 19085176

[15] Shauly O, Gould DJ, Sahai-Srivastava S, Patel KM. Greater Occipital nerve block for the treatment of chronic migraine headaches: a systematic review and Meta-analysis. Plast Reconstr Surg. 2019;144(4):943–52. 31568309 10.1097/PRS.0000000000006059

[16] Tang Y, Kang J, Zhang Y, Zhang X. Influence of greater occipital nerve block on pain severity in migraine patients: a systematic review and meta-analysis. Am J Emerg Med. 2017;35(11):1750–4. 28844531 10.1016/j.ajem.2017.08.027

[17] Zhang H, Yang X, Lin Y, Chen L, Ye H. The efficacy of greater occipital nerve block for the treatment of migraine: a systematic review and meta-analysis. Clin Neurol Neurosurg. 2018;165:129–33. 29421172 10.1016/j.clineuro.2017.12.026

[18] Page MJ, McKenzie JE, Bossuyt PM, Boutron I, Hoffmann TC, Mulrow CD, et al. The PRISMA 2020 statement: an updated guideline for reporting systematic reviews. BMJ. 2021;372:n71. 33782057 10.1136/bmj.n71 PMC8005924

[19] Inan LE, Inan N, Karadaş Ö, Gül HL, Erdemoğlu AK, Türkel Y, et al. Greater occipital nerve blockade for the treatment of chronic migraine: a randomized, multicenter, double-blind, and placebo-controlled study. Acta Neurol Scand. 2015;132(4):270–7. 25765043 10.1111/ane.12393

[20] Gul HL, Ozon AO, Karadas O, Koc G, Inan LE. The efficacy of greater occipital nerve blockade in chronic migraine: a placebo-controlled study. Acta Neurol Scand. 2017;136(2):138–44. 27910088 10.1111/ane.12716

[21] Palamar D, Uluduz D, Saip S, Erden G, Unalan H, Akarirmak U. Ultrasound-guided greater occipital nerve block: an efficient technique in chronic refractory migraine without aura? Pain Physician. 2015;18(2):153–62. 25794201 10.36076/ppj/2015.18.153

[22] Özer D, Bölük C, Türk Börü Ü, Altun D, Taşdemir M, Köseoğlu Toksoy C. Greater occipital and supraorbital nerve blockade for the preventive treatment of migraine: a single-blind, randomized, placebo-controlled study. Curr Med Res Opin. 2019;35(5):909–15. 30285507 10.1080/03007995.2018.1532403

[23] Chowdhury D, Tomar A, Deorari V, Duggal A, Krishnan A, Koul A. Greater occipital nerve blockade for the preventive treatment of chronic migraine: a randomized double-blind placebo-controlled study. Cephalalgia. 2023;43(2):3331024221143541. 36739512 10.1177/03331024221143541

[24] Velásquez-Rimachi V, Chachaima-Mar J, Cárdenas-Baltazar EC, Loayza-Vidalon A, Morán-Mariños C, Pacheco-Barrios K, et al. Greater occipital nerve block for chronic migraine patients: a meta-analysis. Acta Neurol Scand. 2022;146(2):101–14. 35726455 10.1111/ane.13634

[25] Bovim G, Sand T. Cervicogenic headache, migraine without aura and tension-type headache. Diagnostic blockade of greater occipital and supra-orbital nerves. Pain. 1992;51(1):43–8. 1454403 10.1016/0304-3959(92)90007-X

[26] Allen SM, Mookadam F, Cha SS, Freeman JA, Starling AJ, Mookadam M. Greater Occipital nerve block for Acute Treatment of Migraine Headache: a large Retrospective Cohort Study. J Am Board Fam Med. 2018;31(2):211–8. 29535237 10.3122/jabfm.2018.02.170188

[27] Austin M, Hinson MR. Occipital nerve Block. StatPearls. Treasure Island (FL) ineligible companies. Disclosure: Melissa Hinson declares no relevant financial relationships with ineligible companies.: StatPearls Publishing Copyright © 2024. StatPearls Publishing LLC.; 2024.

[28] Afridi SK, Shields KG, Bhola R, Goadsby PJ. Greater occipital nerve injection in primary headache syndromes–prolonged effects from a single injection. Pain. 2006;122(1–2):126–9. 16527404 10.1016/j.pain.2006.01.016

[29] Santos Lasaosa S, Cuadrado Pérez ML, Guerrero Peral AL, Huerta Villanueva M, Porta-Etessam J, Pozo-Rosich P, et al. Consensus recommendations for anaesthetic peripheral nerve block. Neurologia. 2017;32(5):316–30. 27342391 10.1016/j.nrl.2016.04.017

[30] Rothrock JF. Occfipital nerve blocks. Headache the Journal of Head and Face Pain [Internet]. 2010;50(5):917–8. 10.1111/j.1526-4610.2010.01668.x 10.1111/j.1526-4610.2010.01668.x 20546326

[31] Blumenfeld A, Ashkenazi A, Napchan U, Bender SD, Klein BC, Berliner R et al. Expert Consensus Recommendations for the performance of Peripheral nerve blocks for headaches – A Narrative review. Headache the Journal of Head and Face Pain [Internet]. 2013;53(3):437–46. 10.1111/head.12053 10.1111/head.12053 23406160

[32] Ashkenazi A, Matro R, Shaw JW, Abbas MA, Silberstein SD. Greater occipital nerve block using local anaesthetics alone or with triamcinolone for transformed migraine: a randomised comparative study. J Neurol Neurosurg Psychiatry. 2008;79(4):415–7. 17682008 10.1136/jnnp.2007.124420

[33] Dilli E, Halker R, Vargas B, Hentz J, Radam T, Rogers R, et al. Occipital nerve block for the short-term preventive treatment of migraine: a randomized, double-blinded, placebo-controlled study. Cephalalgia. 2015;35(11):959–68. 25505035 10.1177/0333102414561872

[34] Cuadrado ML, Aledo-Serrano Á, Navarro P, López-Ruiz P, Fernández-de-Las-Peñas C, González-Suárez I, et al. Short-term effects of greater occipital nerve blocks in chronic migraine: a double-blind, randomised, placebo-controlled clinical trial. Cephalalgia. 2017;37(9):864–72. 27296456 10.1177/0333102416655159

[35] Karaoğlan M, Durmuş İE, Küçükçay B, Takmaz SA, İnan LE. Comparison of the clinical efficacy of bilateral and unilateral GON blockade at the C2 level in chronic migraine. Neurol Sci. 2022;43(5):3297–303. 34791570 10.1007/s10072-021-05739-5

